# Supplementary material for: Addressing sociodemographic disparities in COVID-19 vaccine uptake among youth in Zimbabwe
Source: BMJ Glob Health. 2023 Jul 5;8(7):e012268. doi: 10.1136/bmjgh-2023-012268 (PMC10335587; doi:10.1136/bmjgh-2023-012268)
Supplement: Supplementary data [file bmjgh-2023-012268supp001.pdf]

Supplemental Table 1: Reason for not getting vaccinated stratified by socioeconomic quintile

	Poorest 20% (n=1897)	2nd quintile (n=1341)	3rd quintile (n=1399)	4th quintile (n=1322)	Least poor 20% (n=1062)
I didn't know that there was a vaccine against COVID-19	15 (0.8%)	9 (0.7%)	12 (0.9%)	11 (0.8%)	5 (0.5%)
I think I'm not eligible for the COVID-19 vaccine	136 (7.2%)	111 (8.3%)	127 (9.1%)	106 (8.0%)	70 (6.6%)
I didn't know where I can get the COVID-19 vaccine	22 (1.2%)	24 (1.8%)	27 (1.9%)	25 (1.9%)	21 (2.0%)
I do not have money for transport to get to the vaccination centre	17 (0.9%)	22 (1.6%)	17 (1.2%)	16 (1.2%)	7 (0.7%)
I do not have time to go and get the vaccine	328 (17.3%)	256 (19.1%)	286 (20.4%)	280 (21.2%)	246 (23.2%)
I have already had COVID-19, so I don't need to be vaccinated	1 (0.1%)	3 (0.2%)	1 (0.1%)	11 (0.8%)	1 (0.1%)
I have heard there are not enough vaccines (stockouts/ restricted supplies) in the country	62 (3.3%)	48 (3.6%)	48 (3.4%)	48 (3.6%)	41 (3.9%)
I went to the vaccination centre but couldn't get the vaccine (not available or long queue)	125 (6.6%)	111 (8.3%)	117 (8.4%)	118 (8.9%)	77 (7.3%)
I am young and healthy, so I don't need a vaccine	80 (4.2%)	78 (5.8%)	96 (6.9%)	117 (8.9%)	83 (7.8%)
The vaccine might hurt my fertility	201 (10.6%)	112 (8.4%)	151 (10.8%)	126 (9.5%)	87 (8.2%)
I am worried about side effects (other than fertility)	377 (19.9%)	228 (17.0%)	265 (18.9%)	254 (19.2%)	207 (19.5%)
I don't think the vaccine is safe	362 (19.1%)	223 (16.6%)	288 (20.6%)	279 (21.1%)	206 (19.4%)
I don't believe the vaccine works	207 (10.9%)	155 (11.6%)	176 (12.6%)	194 (14.7%)	139 (13.1%)
My religion does not allow me to have the COVID-19 vaccine	75 (4.0%)	43 (3.2%)	55 (3.9%)	44 (3.3%)	28 (2.6%)
Afraid of dying after 2 years	300 (15.8%)	190 (14.2%)	216 (15.4%)	196 (14.8%)	125 (11.8%)

Supplemental Table 2: Univariable logistic regression analysis of the association between COVID-19 vaccination and sociodemographic variables

	**Overall OR (95%CI)**	**Harare OR (95%CI)**	**Bulawayo OR (95%CI)**	**Mashonaland East OR (95%CI)**
Sex				
Female	1 (base)	1 (base)	1 (base)	1 (base)
Male	1.83 (1.72 – 1.95)	1.68 (1.51 – 1.88)	1.52 (1.36 – 1.70)	2.05 (1.82 – 2.30)
Age				
< 20	1 (base)	1 (base)	1 (base)	1 (base)
20-22	1.18 (1.10 – 1.28)	1.38 (1.21 – 1.58)	1.36 (1.19 – 1.56)	1.06 (0.92 – 1.21)
> 22	1.22 (1.14 – 1.31)	1.57 (1.39 – 1.78)	1.44 (1.26 – 1.63)	1.05 (0.93 – 1.20)
Education				
Primary	1 (base)	1 (base)	1 (base)	1 (base)
Form 4	1.78 (1.39 – 2.28)	1.79 (1.14 – 2.79)	1.89 (1.19 – 3.00)	1.44 (0.92 – 2.26)
Form 6	3.80 (2.92 – 4.95)	4.54 (2.85 – 7.23)	3.74 (2.29 – 6.11)	3.17 (1.96 – 5.14)
Tertiary	4.71 (3.57 – 6.21)	6.15 (3.82 – 9.89)	4.94 (2.92 – 8.35)	3.98 (2.39 – 6.61)
Socioeconomic status				
Poorest	1 (base)	1 (base)	1 (base)	1 (base)
2nd poorest	1.21 (1.10 – 1.32)	1.38 (1.16 – 1.64)	1.19 (1.02 – 1.39)	1.01 (0.86 – 1.19)
3rd poorest	1.38 (1.26 – 1.51)	1.72 (1.46 - 2.03)	1.20 (0.95 – 1.50)	1.21 (1.02 – 1.42)

4th poorest	1.54 (1.41 – 1.69)	2.28 (1.94 – 2.69)	1.36 (1.12 – 1.59)	1.39 (1.15 – 1.67)
Least poor	1.98 (1.80 – 2.18)	2.86 (2.42 – 3.38)	1.51 (1.27 – 1.80)	2.02 (1.69 – 2.43)

Adjusted by month of recruitment, OR=odds ratio, 95%CI=95% confindence interval

Supplemental Table 3: Multivariable logistic regression analysis of the association between COVID-19 vaccination and sociodemographic variables in the control clusters of the CHIEDZA trial

	**Overall (n=8,788) OR (95% CI)**	**Harare (n=2,918) OR (95% CI)**	**Bulawayo (n=2,976) OR (95% CI)**	**Mashonaland East (n=2,894) OR (95% CI)**
Sex				
Female	1 ($p < 0.0001$)	1 ($p < 0.0001$)	1 ($p = 0.0051$)	1 ($p < 0.0001$)
Male	1.66 (1.51 – 1.82)	1.59 (1.35 – 1.86)	1.26 (1.07 – 1.49)	2.16 (1.82 – 2.56)
Age				
< 20	1 ($p = 0.0027$)	1 ($p = 0.0026$)	1 ($p = 0.0029$)	1 ($p = 0.2994$)
20-22	1.12 (1.00 – 1.25)	1.25 (1.02 – 1.52)	1.23 (1.01 – 1.50)	0.96 (0.78 – 1.18)
> 22	1.20 (1.08 – 1.33)	1.37 (1.14 – 1.64)	1.39 (1.14 – 1.68)	1.12 (0.93 – 1.36)
Education				
Primary	1 ($p < 0.0001$)	1 ($p < 0.0001$)	1 ($p < 0.0001$)	1 ($p < 0.0001$)
Form 4	1.59 (1.41 – 1.79)	1.62 (1.31 – 2.01)	1.78 (1.44 – 2.20)	1.42 (1.16 – 1.74)
Form 6	3.17 (2.65 – 3.80)	3.19 (2.36 – 4.32)	3.36 (2.45 – 4.61)	3.02 (2.13 – 4.28)
Secondary and above	4.40 (3.52 – 5.49)	4.88 (3.44 – 6.92)	5.94 (3.74 – 9.43)	3.15 (2.12 – 4.69)
Socioeconomic status				
Poorest 20%	1 ($p = 0.0151$)	1 ($p = 0.0047$)	1 ($p = 0.5191$)	1 ($p = 0.0014$)
2nd poorest 20%	0.93 (0.81 – 1.07)	1.08 (0.84 – 1.40)	1.16 (0.88 – 1.53)	0.84 (0.67 – 1.06)
3rd poorest 20%	1.05 (0.92 – 1.21)	1.24 (0.97 – 1.59)	1.22 (0.94 – 1.59)	1.09 (0.85 – 1.40)

4th poorest 20%	1.06 (0.93 – 1.22)	1.31 (1.02 – 1.67)	1.21 (0.93 – 1.58)	1.13 (0.88 – 1.46)
Least poor	1.21 (1.04 – 1.40)	1.60 (1.23 – 2.08)	1.07 (0.80 – 1.41)	1.56 (1.19 – 2.05)

Analysis was adjusted for month of recruitment. OR=odds ratio, 95% CI=95% confidence interval. P-values are the result of a likelihood ratio test

BMJ Publishing Group Limited (BMJ) disclaims all liability and responsibility arising from any reliance placed on this supplemental material which has been supplied by the author(s)

Supplemental Table 4: List of assets used in the principal component analysis to derive socioeconomic status

Assets	Response options
Refrigerator	Yes/No
Bicycle	Yes/No
Car	Yes/No
Television	Yes/No
Radio	Yes/No
Microwave	Yes/No
Cell phone	Yes/No
Computer	Yes/No
